# Mapping of QTL for Fusarium head blight resistance and morphological and developmental traits in three backcross populations derived from *Triticum dicoccum* × *Triticum durum*

**DOI:** 10.1007/s00122-012-1951-2

**Published:** 2012-08-25

**Authors:** Maria Buerstmayr, Karin Huber, Johannes Heckmann, Barbara Steiner, James C. Nelson, Hermann Buerstmayr

**Affiliations:** 1 Department for Agrobiotechnology Tulln, BOKU-University of Natural Resources and Life Sciences-Vienna, Konrad Lorenz Str. 20, 3430 Tulln, Austria; 2 Department of Plant Pathology, Kansas State University, Manhattan, KS 66506 USA; 3 Present Address: Rijk Zwaan Nederland B.V., Burgemeester Crezéelaan, P.O. Box 40, 2678 ZG De Lier, The Netherlands; 4 Present Address: Presse und Informationsdienst Agrarisches Informationszentrum (AIZ), Schauflergasse 6, 1014 Vienna, Austria

## Abstract

Breeding for resistance to *Fusarium* head blight (FHB) in durum wheat continues to be hindered by the lack of effective resistance sources. Only limited information is available on resistance QTL for FHB in tetraploid wheat. In this study, resistance to FHB of a *Triticum dicoccum* line in the background of three Austrian *T. durum* cultivars was genetically characterized. Three populations of BC_1_F_4_-derived RILs were developed from crosses between the resistant donor line *T. dicoccum*-161 and the Austrian *T. durum* recipient varieties DS-131621, Floradur and Helidur. About 130 BC_1_F_4_-derived lines per population were evaluated for FHB response using artificial spray inoculation in four field experiments during two seasons. Lines were genetically fingerprinted using SSR and AFLP markers. Genomic regions on chromosomes 3B, 4B, 6A, 6B and 7B were significantly associated with FHB severity. FHB resistance QTL on 6B and 7B were identified in two populations and a resistance QTL on 4B appeared in three populations. The alleles that enhanced FHB resistance were derived from the *T. dicoccum* parent, except for the QTL on chromosome 3B. All QTL except the QTL on 6A mapped to genomic regions where QTL for FHB have previously been reported in hexaploid wheat. QTL on 3B and 6B coincided with *Fhb1* and *Fhb2*, respectively. This implies that tetraploid and hexaploid wheat share common genomic regions associated with FHB resistance. QTL for FHB resistance on 4B co-located with a major QTL for plant height and mapped at the position of the *Rht*-*B1* gene, while QTL on 7B overlapped with QTL for flowering time.

**Electronic supplementary material:**

The online version of this article (doi:10.1007/s00122-012-1951-2) contains supplementary material, which is available to authorized users.

## Introduction

Fusarium head blight (FHB), caused by several members of the *Fusarium* genus, occurs frequently in small grain cereals in temperate regions throughout the world (McMullen et al. 1997). FHB leads to severe losses not only in grain yield but also in quality; contamination with toxic fungal metabolites such as deoxynivalenol or nivalenol render harvested grain unsuitable for consumption as food and feed (Gilbert and Tekauz 2000). This contamination is especially critical for durum wheat, which is used primarily for human consumption. The best economic and ecological strategy for reducing FHB damage is the utilization of resistant cultivars.

Although a range of *Fusarium* species cause FHB and different *Fusarium* strains may differ widely in aggressiveness, no biological races with a specific host–pathogen interaction have been reported (Van Eeuwijk et al. 1995). Resistance to FHB is complex and quantitatively inherited; infection and development of FHB depend largely on environment, and genotype × environment interaction complicates resistance evaluation (Miedaner et al. 2001). Flowering time is the most sensitive plant development stage for FHB infection (Atanasoff 1920; Parry et al. 1995); warm temperature (Andersen 1948; Parry et al. 1995) and high humidity during flowering promote infection (Cook 1981). Both active resistance factors, which include physiological processes (Crute et al. 1985), and passive factors, such as plant height, spike architecture and flowering date, influence infection and/or disease development (Buerstmayr et al. 2009; Mesterhazy 1995) and it may be difficult to dissect resistance factors. Thus, considering these plant traits in the analysis of FHB resistance is important. The distinction between resistance to initial infection (type 1) and resistance to fungal spread of the pathogen within the spike (type 2) first described by Schroeder and Christensen (1963) is widely accepted. These resistance types can be distinguished by quantitative trait loci/locus (QTL) studies employing suitable inoculation and assessment methods (Buerstmayr et al. 2009).

In bread wheat numerous QTL have been described, for example the major *Fhb1* QTL on chromosome 3BS and *Fhb2* on chromosome 6BS have repeatedly been found in independent QTL studies (Buerstmayr et al. 2009). QTL corresponding to the *Fhb2* locus were detected in tetraploid wheat as well (Somers et al. 2006). But of 52 QTL studies reviewed by Buerstmayr et al. (2009), only four concern resistance sources of tetraploid wheat. Although an extensive collection of about 6,000 durum wheat accessions were screened for FHB resistance, none showed enhanced resistance, and a further screening survey of material from CIMMYT and ICARDA identified only five lines—from a Tunisian source—that exhibited moderate resistance to FHB spread (Elias et al. 2005; Huhn et al. 2012). It was accordingly speculated that durum wheat either lacks resistance genes or carries effective susceptibility factors and/or suppressor genes that compromise FHB resistance (Ban and Watanabe 2001; Kishii et al. 2005). Indeed, a QTL that increased FHB susceptibility was reported at chromosome 2A of the *T. dicoccoides* line Israel A (Garvin et al. 2009). Fakhfakh et al. (2011) hypothesized that the D genome of hexaploid wheat encodes resistance-inducing factors that are missing in tetraploid wheat. Gilbert et al. (2000) studied the influence of the D genome on F_1_ and F_2_ pentaploid plants of crosses from resistant lines of Sumai-3, Ning8331 and 93FHB21 to the susceptible tetraploids Stewart 63 and DT486, but they did not find a relationship between the presence/absence of D chromosomes and FHB reaction.

Durum wheat accounts for only 4 % of total wheat production worldwide (Gill et al. 2004) so that activity in durum wheat improvement is lower than in bread wheat. The tetraploidy of durum wheat and limited breeding efforts in this relatively recent crop may have led to a narrow genetic base compared to hexaploid wheat (Oliver et al. 2008). Attempts to transfer resistance from hexaploid into tetraploid wheat have met with limited success (Gilbert et al. 2000; Oliver et al. 2007; authors’ unpublished results). For this reason, studies have been conducted to find resistance sources in cultivated or wild relatives of durum wheat (Buerstmayr et al. 2003; Clarke et al. 2004; Kishii et al. 2005; Miller et al. 1998; Oliver et al. 2004, 2007, 2008). Several moderately FHB-resistant accessions of wild emmer wheat, *T. dicoccoides* (Buerstmayr et al. 2003; Miller et al. 1998; Oliver et al. 2007), cultivated emmer wheat, *T. dicoccum*, and Persian wheat, *T*. *carthlicum* (Oliver et al. 2008), have been identified. Three sets of disomic chromosome substitution lines derived from *T. dicoccoides* accessions (Israel A, PI478742 and PI481521) in the genetic background of the *T. durum* cultivar Langdon (LDN) (Joppa and Williams 1988; Kumar et al. 2007) were tested for FHB response (Stack et al. 2002). Subsequent studies mapped QTL for FHB resistance derived from *T. dicoccoides* accession Israel A on chromosome 3A near *Xgwm2* (Chen et al. 2007; Otto et al. 2002), and on the short arm of chromosome 6B (Stack and Faris 2006). The resistance of *T. dicoccoides* accession PI478742 on chromosome 7A could be assigned to 7AL (Kumar et al. 2007). In a BC_1_-derived RIL population from the cross of *T. dicoccoides* accession Mt. Hermon #22 with the *T. durum* cultivar Helidur, four QTL were discovered and mapped to chromosomes 3A, 4A (with the resistant allele from *T. dicoccoides*), 2B and 4B (resistant allele from *T. durum*). The QTL with the largest effect was identified on 3A near *Xgwm2* (Gladysz et al. 2007) and colocalizes with the QTL derived from the resistance source of Israel A. In a doubled-haploid population from a cross of the *T. durum* cultivar Strongfield with the *T. carthlicum* cultivar Blackbird, two significant QTL for FHB spread within the spike were found, mapping to chromosome arms 2BL and 6BS (Somers et al. 2006). Notably, the 6BS QTL derived from Blackbird appeared to coincide with *Fhb2* and the QTL on chromosome 2BL derived from durum parent Strongfield covers the same genomic region as the QTL of the *T. durum* cultivar Helidur. Ghavami et al. (2011) used breeding populations derived from several crosses of moderately resistant Tunisian durum wheat accessions with North Dakota durum lines for bi-parental and association mapping. They discovered a consistent type 2 resistance QTL at chromosome arm 5BL, at which, interestingly, the resistance-improving allele derived from the moderately susceptible *T. durum* cultivar Lebsock and not the Tunisian durum parent.

The most FHB-resistant tetraploid wheat line tested so far at IFA Tulln (Austria) has been *T. dicoccum* line 161 (hereafter Td161). This line shows a remarkable level of FHB resistance in replicated experiments, both after single-spikelet inoculation and after spray inoculation in field and greenhouse experiments (unpublished results). The objective of the present investigation was to dissect the FHB resistance of Td161 genetically and to study the influence of plant architecture and flowering date on disease development. For this purpose we generated three BC_1_F_4_ populations from crosses of Td161 with three different well adapted but FHB-susceptible Austrian *T. durum* cultivars. The aim of a backcross step to the respective *T. durum* parents was to provide an additional round of recombination and to increase the proportion of the overall genome of the *T. durum* parents, so that resistance from *T. dicoccum* could be tested in an agronomically acceptable genetic background. Using spray inoculation, we evaluated the overall FHB resistance conferred by Td161 simultaneously in the genetic background of these three modern durum cultivars, allowing detection, comparison and validation of the effectiveness of FHB resistance QTL.

## Materials and methods

### Plant material

The Fusarium-resistant homozygous *T. dicoccum* line Td161 and three susceptible *T. durum* wheat varieties were used to generate three populations segregating for FHB resistance. Td161 was provided by Dr. Jeannie Gilbert (Agriculture and Agri-Food Canada, Winnipeg). Td161 is a hulled wheat, has a long and dense-spike phenotype, and is tall with a tendency to lodging. The Austrian *T. durum* breeding line DS-131621 (abbreviated DS) with pedigree CIMMYT-4833//Cando/Valgerado and *T. durum* cultivars Floradur with pedigree Helidur/CIMMYT-4833 and Helidur with pedigree Pandur/CPB132/3/Valdur//Pandur/Valgerado were used as the recurrent parents. The *T. durum* parents were provided by Saatzucht-Donau, Austria. In contrast to Td161, the recurrent parents are relatively short (carrying the *Rht*-*B1b* allele for reduced plant height) and possess a dense-spike phenotype. Typical heads of Td161, Helidur and Floradur are shown in Electronic Supplementary Material Fig. S1. F_1_ plants from each cross were backcrossed as the female to their respective *T. durum* parent. BC_1_F_1_ plants were advanced by single-seed descent to the BC_1_F_4_ generation. The resulting BC_1_F_4_ plants were bulk propagated for multi-environment testing as BC_1_F_4:5_ lines. The three BC_1_F_4_ populations, here abbreviated as DTd (recurrent parent DS-131621), FTd (Floradur) and HTd (Helidur), comprised 134, 129 and 126 BC_1_F_4_ lines, respectively.

### Field experiments and disease assessment

All populations were tested in four field experiments at IFA-Tulln, 30 km west of Vienna (16°04’E, 48°19’N, 177 m above sea level) in 2006 and 2008. In each year two experiments were conducted, one inoculated with *F. culmorum* (Fc) and the other with *F. graminearum* (Fg). Accordingly, experiments are encoded by isolate and year as Fc06, Fc08, Fg06 and Fg08. Experiments were arranged in a randomized complete block design with two blocks. Plots consisted of double rows of 1 m length and 17 cm spacing. In 2006 the sowing time was early spring. The two replications were sown 1 week apart. One replication of the 2008 experiment was sown in November 2007 and the second in early spring 2008. These staggered sowing dates led to slightly different flowering dates between the blocks. Crop management was essentially as described by Buerstmayr et al. (2002). All experiments were spray inoculated with a motor-driven backpack sprayer in the late afternoon. Each plot was individually inoculated twice, the first time when 50 % of the heads within a plot were flowering and the second time 2 days later. Plots were mist irrigated for 20 h after inoculation to facilitate infection. For inoculation, macroconidial suspensions of either *F. culmorum* single-spore isolate ‘IFA-106’, prepared as described by Buerstmayr et al. (2000), or *F. graminearum* single-spore isolate ‘IFA-65’, prepared as described by Buerstmayr et al. (2003), were used. Aliquots of conidia stock solutions were stored at −30 °C and diluted with deionized water to a final spore concentration of 2.5 × 10^4^mL^−1^ just prior to inoculation. FHB severity was averaged as the visually estimated percentage of infected spikelets per plot on days 10, 14, 18, 22 and 26 after first inoculation. This inoculation and scoring method mimics a natural epidemic and reflects overall resistance, but does not distinguish specific types of resistance (Buerstmayr et al. 2009). Date of anthesis was recorded for each plot and converted into number of days after May 1. Plant height was measured for experiments Fc06 and Fg08 in cm. Awn length was visually scored from 0 (short) to 9 (long) in experiments Fc08 and Fg08. Spike density was scored from 0 (loose) to 9 (very compact) in experiment Fg08. Awn length and ear compactness were visually assessed in the field after anthesis.

### Molecular genetic characterization

117 lines of population DTd and 120 lines of population FTd and 120 lines of population HTd were randomly chosen for marker analysis. Total genomic DNA was isolated from young leaves of 10 pooled plants of each backcross line and of the parental lines according to the protocol of Saghai Maroof et al. (1984). All populations were genotyped with simple sequence repeat (SSR) and amplified fragment length polymorphism (AFLP) markers and allele-specific SNP markers for *Rht*-*B1a* (tall) and *Rht*-*B1b* (short) (Ellis et al. 2002). A polymorphism survey on the parents was carried out with 237 SSR primer pairs, comprising 158 GWM markers (Roeder et al. 1998), 71 BARC markers (Song et al. 2005), 6 WMC markers (Somers et al. 2004), 1 GDM marker (Pestsova et al. 2000), the *umn10* marker (Liu et al. 2008) and allele-specific *Rht*-*B1* markers (Ellis et al. 2002). From these primer pairs 85 were chosen for screening the DTd, FTd and HTd population, respectively. PCR and fragment detection were conducted as described by Steiner et al. (2004). AFLP marker analysis (Vos et al. 1995) was performed using *Mse*I/*Sse*8387I restriction enzymes as described by Hartl et al. (1999) and Buerstmayr et al. (2002). For populations DTd, FTd, and HTd, 21, 24 and 32 selective AFLP primer combinations were used, respectively. For populations FTd and HTd, detection of AFLP fragments was carried out on a LI-COR 4200 dual-dye DNA analyzer (LI-COR Biosciences, Lincoln, Nebraska, USA), and for population DTd a Typhoon-TRIO fluorescence scanner (GE Healthcare, http://www.gehealthcare.com) was used. AFLP markers were abbreviated according to the standard list for AFLP primer nomenclature (http://wheat.pw.usda.gov/ggpages/keygeneAFLPs.html) followed by the starting character of the *T. durum* recurrent-parent name and a number assigned to each unique polymorphic locus. Identical AFLP loci of two or all three populations were encoded by the starting character and corresponding locus number of the respective population.

The *T. durum* parents and Td161 together with hexaploid wheat Sumai-3, CM-82036 and W14 were genotyped with selected markers close to *Fhb1* (*umn10, barc133, gwm533, gwm493, gwm133, barc147*) and *Fhb2* (*wmc397, wmc398, gwm644*) loci. All three hexaploid wheats carry the *Fhb1* resistance allele, and Sumai-3 and W14 also carry the *Fhb2* resistance allele.

### Statistical analysis

#### Field data

Area under the disease progress curve (AUDPC), calculated according to Buerstmayr et al. (2000), was used as a measure of FHB severity. Pearson correlation coefficients between the recorded traits were estimated based on mean across experiments, and for FHB severity, between each experiment combination. The effects of replication within experiments, experiment, genotype, and genotype-by-experiment interaction were estimated using the general linear model (GLM) procedure, with all effects fixed. For the estimation of variance components and broad-sense heritability all effects were considered random. Broad-sense heritability was estimated from variance components with the equation *H* ^2^ = σ_G_^2^/(σ_G_^2^ + σ_G×E_^2^/*e* + σ_E_^2^/*en*), where σ_G_^2^ = genotypic variance, σ_GxE_^2^ = genotype-by-experiment interaction variance, σ_E_^2^ = error variance, *e* = number of experiments and *n* = number of replications (Nyquist 1991). ANOVA and correlation analysis were calculated in SAS/STAT version 9.2 (SAS Institute Inc 2008).

#### Linkage mapping

Segregation deviation of individual markers from expected ratios was determined by Chi-square tests. All linkage maps were constructed using CarthaGène 1.2-LKH for Linux (de Givry et al. 2005) specifying a BC_1_F_4_ genetic model. First, genetic maps for the three populations were calculated independently. A maximum distance of 30 centimorgans (cM) and a minimum logarithm of odds (LOD) threshold of 3 were used to partition markers into linkage groups. The most likely positions of the markers along the linkage groups were determined using the commands *nicemapl*, *mfmapl*, *flips*, *build*, and *annealing*. Cosegregating markers were merged into single markers. Colinearity of the three maps was visualized using MapChart v2.2 (Voorrips 2002) via SSR markers and co-located AFLP markers. These improved data sets were subsequently used for a joint analysis of all populations with CarthaGène. The three data sets (populations) were merged using the command *dsmergor*. This produces consensus data sets sharing marker order, but separate parameter estimates with per-data-set distances (CarthaGène user manual). For calculating cM distances the Kosambi mapping function was used. Linkage groups were assigned to chromosomes according to SSR markers and their map information from GrainGenes (http://wheat.pw.usda.gov/ggpages/maps.shtml). Maps were compared to the high-density wheat consensus SSR genetic map (Somers et al. 2004) available in GrainGenes.

#### QTL mapping

Quantitative trait loci calculations were carried out with R version 2.12.2 (R Development Core Team 2011) based on QTL expectations calculated at 2 cM intervals with QGene 4.3.8 (Joehanes and Nelson 2008) from marker data and map information.

Linear models were fitted to estimate QTL effects on the analyzed traits. For trait FHB severity (AUDPC) the mean temperature over 4 days after first inoculation was included as a covariable. QTL for FHB severity were fitted individually for each experiment using the model *T*_*i*_ = *μ* + *M*_*i*_ + *t* + *ε*_*i*_, where *μ* = general mean, *M*_*i*_ = expected genotype of *i*th QTL, *t* = effect of temperature at flowering, *ε*_*i*_ = random error. A multienvironment analysis was performed including all experiments, using the model *T*_*ijk*_ = *μ* + *M*_*i*_ + *Y*_*j*_ + *I*_*k*_ + *t*_*jk*_ + ε_*ijk*_, where *Y*_*j*_ = effect of *j*th year, *I*_*k*_ = effect of *k*th isolate, *t*_*jk*_ = effect of temperature at flowering in the *j*th year with the *k*th isolate. QTL of morphological traits were calculated using the simplified model *T*_*i*_ = *μ* + *M*_*i*_ + *ε*_*i*_ and, for developmental-trait flowering time, the model *T*_*ij*_ = *μ* + *M*_*i*_ + *Y*_*j*_ + *ε*_*ij*_. *F* statistics were converted into LOD values and the associated explained phenotypic variances were calculated. Additive effects were estimated as the regression coefficients for the corresponding *M*_*i*_ terms.

For all analyses, LOD significance thresholds for type I error rates of α < 0.1, α < 0.05 and α < 0.01 were determined via 1,000 permutations. Linkage groups and LOD bars were drawn with MapChart v2.2.

## Results

### Trait variation

**Fig. 1:**
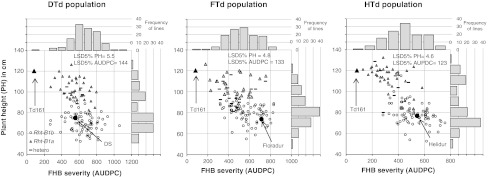
Scatterplots of overall means for FHB AUDPC against plant height with marginal histograms of their frequency distribution. Allele status of *Rht*-*B1* of individual lines is represented by *different symbols*. *Arrows* indicate position of parents

Mean values of the parents, means and ranges of the populations, least significant differences and broad-sense heritability for FHB severity (AUDPC) and for several morphological and developmental traits are summarized in Table 1. All populations displayed continuous distributions of AUDPC. None of the lines exhibited higher resistance than the resistant parent, but several lines from each population showed (*p* < 0.05) higher FHB severity than the susceptible parent (Fig. 1). The average FHB severity of the three populations was lowest in HTd, followed by FTd, and was highest in DTd. Generally experiments inoculated with *F. culmorum* had two- to threefold higher disease severity than those inoculated with *F. graminearum*. Correlation coefficients (*r*) for AUDPC between averaged values of Fg and Fc experiments were high with *r* = 0.66 for DTd and *r* = 0.77 for FTd and HTd. Correlations between individual experiments were all positive and highly significant (*p* < 0.001) and showed ranges *r* = 0.44–0.53, 0.37–0.63, and 0.50–0.65 for the DTd, FTd, and HTd populations, respectively.

**Table 1:** Means of parents, mean, minimum and maximum values of populations, least significant differences at α < 0.05 (LSD) and broad-sense heritability (*H* ^2^) or repeatability of analyzed traits

	Parents				Population				
					DTd				
	Td161	DS	Floradur	Helidur	Mean	Min	Max	LSD5 %	*H* ^2^
FHB severity (AUDPC)									
Overall mean	87	582	710	544	618	254	1,063	144	0.76
Mean *F. culm*	123	862	1,008	816	898	295	1,356	176	0.65
Mean *F. gram*	51	302	413	273	339	120	771	103	0.58
Fc06	109	655	1,007	725	886	125	1,401	189	0.63^d^
Fc08	138	1,069	1,009	906	910	255	1,542	165	0.70^d^
Fg06	17	244	471	224	362	65	830	130	0.65^d^
Fg08	86	360	355	323	315	164	730	68	0.68^d^
Flowering date^a^	51	42.6	41.2	39.1	41.2	38.3	49.0	0.89	0.94
Plant height (cm)	120.3	75.0	73.9	76.9	80.0	47.5	126.3	5.47	0.98
Spike compactness^b^	1.0	5.0	5.0	6.0	4.6	1.0	9.0	1.20	0.67^d^
Awn length^c^	0.0	9.0	9.0	9.0	7.4	0.0	9.0	0.99	0.88^d^

	Population									
	FTd					HTd				
	Mean	Min	Max	LSD5 %	*H* ^2^	Mean	Min	Max	LSD5 %	*H* ^2^
FHB severity (AUDPC)										
Overall mean	534	179	834	133	0.77	448	130	760	123	0.76
Mean *F. culm*	776	289	1,250	161	0.60	671	153	1,143	160	0.69
Mean *F. gram*	292	69	619	99	0.64	225	57	469	70	0.68
Fc06	813	213	1,360	159	0.74^d^	633	80	1,239	152	0.78^d^
Fc08	739	222	1,470	165	0.69^d^	709	226	1,296	169	0.64^d^
Fg06	277	43	779	110	0.65^d^	206	31	549	80	0.70^d^
Fg08	306	95	665	86	0.48^d^	243	67	429	58	0.57^d^
Flowering date^a^	42.0	38.0	57.0	0.96	0.94	40.4	38.0	47.3	0.88	0.95
Plant height (cm)	88.4	55.0	126.3	4.77	0.95	91.0	52.5	132.5	4.55	0.98
Spike compactness^b^	4.1	1.0	7.0	1.18	0.60^d^	4.3	1.0	9.0	1.61	0.30^d^
Awn length^c^	7.7	0.0	9.0	0.97	0.88^d^	7.7	1.0	9.0	0.97	0.85^d^

^a^Number of days from May 1st to mid-anthesis

^b^Visually scored 0 = loose spike to 9 = very compact spike

^c^Visually scored 0 = short awns to 9 = long awns

^d^Repeatability, means based on two replications

Averaged across all experiments, the respective *T. durum* parents DS, Floradur and Helidur were 45, 46, and 44 cm shorter and flowered 9, 10, and 12 days earlier than the donor parent Td161. Compared to the highly FHB-resistant *T. aestivum* line CM-82036, which was included in all experiments as resistant check, Td161 was flowering 2 weeks later, was 35 cm taller, and had an average FHB severity at 26 days after inoculation of 12 % while CM-82036 had 5 %. All populations showed significant variation for the developmental trait flowering time and for the morphological traits plant height, spike compactness and awn length (Table 1). A bimodal frequency distribution for plant height was apparent in populations DTd and HTd (Fig. 1). All populations showed highly significant correlations between FHB severity and plant height (Table 2), which was highest (*r* = −0.70) in HTd. FHB severity, averaged across all experiments, was negatively correlated with flowering time in all populations (Table 2), but flowering time had no association with FHB severity in several individual experiments (Fg08 in population DTd, Fc06 in FTd, Fc06 and Fg08 in HTd). A positive correlation between spike compactness and FHB severity was evident in FTd and HTd. Taller plants as well as earlier-flowering lines were less infected and lines with compact spikes tended to be more infected. Awn length had a weak negative correlation with FHB severity in population HTd, with longer awns slightly decreasing FHB severity.

**Table 2:** Pearson correlation coefficients between line mean values of FHB severity (AUDPC) and morphological traits

	FHB severity measured in AUDPC		
	DTd	FTd	HTd
Flowering date	−0.28**	−0.34***	−0.17*
Plant height	−0.40***	−0.39***	−0.70***
Spike compactness	0.14	0.24**	0.21*
Awn length	0.13	0.06	−0.18*

* *p* < 0.05

** *p* < 0.01

*** *p* < 0.001

ANOVA for FHB severity (as AUDPC) yielded highly significant effects for all sources of variance (Table 3). Broad-sense heritabilities for means over all experiments were constant among the populations with *H* ^2^ = 0.76–0.77 (Table 1). Higher mean temperature during 4 days after first inoculation increased infection (*p* < 0.001) for experiments Fg06, Fc08, and Fg08 in all populations. To account for this dependence, temperature was incorporated as a co-variable in the QTL analysis model.

**Table 3:** Analysis of variance for FHB severity measured in AUDPC across all experiments

Source	Population								
	DTd			FTd			HTd		
	*df*	Mean square	*F* value	*df*	Mean square	*F* value	*df*	Mean square	*F* value
Blocks within Exp	4	3,440,238	127.4*	4	2,243.458	97.8*	4	968,268	49.6*
Experiment	3	26,997,987	999.8*	3	20,080.798	875.7*	3	16,925,090	867.0*
Genotype	134	183,847	6.8*	129	175.515	7.7*	126	160,476	8.2*
Genotype × Exp.	401	43,462	1.6*	375	40.608	1.8*	376	37,837	1.9*
Error	517	27,002		494	14.214		501	19,522	

Significant at * *p* < 0.001

### Linkage maps

Of the 237 SSR markers tested on the parents 191 (80.6 %) were polymorphic. The SSR and AFLP markers yielded 480, 311 and 295 polymorphic loci in populations DTd, FTd, and HTd. After cosegregating markers were merged into single markers, the final maps comprised 368, 248, and 239 loci, respectively, among which 102 markers mapped across all populations.

The observed allele segregation of the various markers fitted in most cases the expected ratio of a BC_1_F_4_ RIL population. Segregation distortion at *p* < 0.05 was observed for 19 markers in DTd, 26 markers in FTd, and 19 markers in HTd.

The markers of population DTd fell into 38 linkage groups, of which 13 (827 cM) could be assigned to genome A and 13 (858 cM) to genome B, while 12 (236 cM) could not be unambiguously assigned to a chromosome. Markers of population FTd fell into 36 linkage groups, consisting of 15 (606 cM) on genome A, 12 (686 cM) on genome B, and 9 (122 cM) unassigned groups, and for population HTd 32 groups consisted of 14 (621 cM) on genome A, 10 (755 cM) on genome B, and 8 unassigned (139 cM). Total map lengths were 1,921, 1,414, and 1,515 cM for DTd, FTd, and HTd, resulting in average marker distances of 5.2, 5.7 and 6.3 cM. For all chromosomes at least partial maps were obtained.

### Haplotype comparison for SSR markers at Fhb1 (3BS) and Fhb2 (6BS)

An allele survey of Sumai-3, W14, CM-82036, and the parents Td161, DS, Floradur and Helidur with selected markers in the vicinity of *Fhb1* and *Fhb2* showed for all markers different alleles between the hexaploid and tetraploid lines studied. In the region surrounding *Fhb1* all hexaploid wheats shared one common haplotype, the durum parents Floradur and Helidur formed a second haplotype, alleles of DS differed at two markers (*barc133, barc147*) from Floradur and Helidur, and Td161 varied at all marker loci from all analyzed lines, except for *umn10*. All analyzed tetraploid wheat lines/cultivars possessed a null allele at *umn10.* In the *Fhb2* region, the hexaploid wheat lines that carry the *Fhb2* resistance QTL, displayed one haplotype and the durum parents a second haplotype, with only Floradur differing from Helidur and DS by allele size at one marker (*wmc398*). Also at *Fhb2* Td161 carried unique alleles at all tested marker loci.

### QTL analysis

#### Quantitative trait loci analysis for FHB severity

**Fig. 2:**
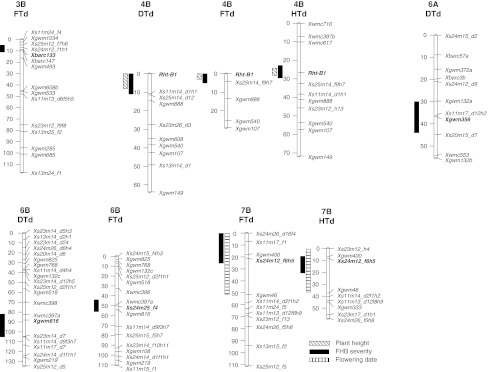
Linkage maps and positions of QTL for FHB severity and QTL of coinciding morphological/developmental traits of the three populations. Loci closest to the QTL peak of FHB severity are in *bold*. QTL bars span a LOD drop of 1.5 from maximum LOD

Quantitative trait loci for FHB severity and their positions and statistical parameters are summarized in Table 4. Only QTL with LOD values >3 in two or more experiments or exceeding in one or more populations the LOD significance threshold for the multienvironment analysis are presented. Among the three populations, five genomic regions, on chromosomes 3B, 4B, 6A, 6B and 7B, were associated with FHB severity (Table 4; Fig. 2, Electronic Supplementary Material Fig. S2). Three of these QTL were detected in two or three populations. Except for QTL on chromosome 3B, the allele that improved resistance was derived from the *T. dicoccum* donor parent Td161.

**Table 4:** Summary of QTL for FHB severity (AUDPC) identified by simple interval mapping

Pop	Chro	Flanking markers	Closest marker	Multienvironment analysis		
				Add^a^	% PV	LOD^b^
FTd	3B	*Xs25m12_f7h6*–*Xbarc147*	*Xbarc133*	−62	5.3	10.7**
DTd	4B	*Rht*-*B1*–*Xs11m14_d1h1*	*Rht*-*B1*	51	3.1	**6.4**
FTd	4B	*Rht*-*B1*–*Xs25m14_f9h7*	*Rht*-*B1*	59	4.9	10.0**
HTd	4B	*Xwmc617*–*Xs25m14_f9h7*	*Rht*-*B1*	101	18.0	39.2***
DTd	6A	*Xgwm132a*–*Xs20m15_d7*	*Xgwm356*	62	4.0	8.3**
DTd	6B	*Xwmc398*–*Xs23m14_d7*	*Xgwm816*	45	2.4	**4.9**
FTd	6B	*Xwmc398*–*Xgwm816*	*Xs24m25_f4*	57	4.8	9.8**
FTd	7B	*Xs24m26_d16f4*–*Xs24m12_f6h5*	*Xs24m12_f6h5*	55	3.3	**6.6**
HTd	7B	*Xgwm400*–*Xgwm46*	*Xs24m12_f6h5*	38	2.2	**4.7**

Pop	Chro	Flanking markers	*F. culmorum*				*F. graminearum*			
			2006		2008		2006		2008	
			% PV	LOD^b^	% PV	LOD^b^	% PV	LOD^b^	% PV	LOD^b^
FTd	3B	*Xs25m12_f7h6*–*Xbarc147*	12.4	6.8***	4.5	2.2	5.2	2.7	9.4	4.7***
DTd	4B	*Rht*-*B1*–*Xs11m14_d1h1*	4.1	2.1	0.2	0.1	11.7	6.3***	8.9	4.7**
FTd	4B	*Rht*-*B1*–*Xs25m14_f9h7*	6.4	**3.4**	4.9	2.4	8.6	4.6*	9.7	4.8***
HTd	4B	*Xwmc617*–*Xs25m14_f9h7*	27.2	16.3***	20.2	11.6***	26.9	16.1***	16.6	9.3***
DTd	6A	*Xgwm132a*–*Xs20m15_d7*	3.1	1.6	10.4	5.5**	5.7	3.0*	5.3	2.7
DTd	6B	*Xwmc398*–*Xs23m14_d7*	1.3	0.7	5.8	**3.0**	3.5	1.8	6.2	**3.2**
FTd	6B	*Xwmc398*–*Xgwm816*	3.8	2.0	12.8	6.5**	3.8	2.0	8.7	4.3**
FTd	7B	*Xs24m26_d16f4*–*Xs24m12_f6h5*	0.2	0.1	19.5	10.2***	8.4	4.5**	0.3	0.1
HTd	7B	*Xgwm400*–*Xgwm46*	0.9	0.5	11.3	6.2**	2.7	1.4	1.3	0.7

LOD values ≥ 3 are printed in **bold**

* α 0.1 < LOD; ** α 0.05 < LOD; *** α 0.01 < LOD

^a^Positive additive effects denote that the *T.diccocum* allele reduces trait values relative to its respective *T. durum* allele

^b^Significance thresholds were estimated by permutation tests (number of iterations = 1,000) for α 0.01, α 0.05, α 0.1 for each experiment and for the multienvironment analysis of all populations

The QTL on chromosome 3B close to *Xbarc133* appeared only in population FTd and was significant in two of the four experiments. For this region, the susceptible *T. durum* parent Floradur contributed the resistant allele. The FHB QTL on chromosome 4B was significant in all three populations and coincided with QTL for plant height at the *Rht*-*B1* locus. This QTL for FHB was observed in *F. graminearum*-inoculated experiments of populations DTd and FTd and in all experiments of HTd. It had the greatest effect on FHB in population HTd, where it explained 18 % of the phenotypic variance (PV) in the multienvironment analysis. Populations DTd and FTd showed a QTL in the same region on chromosome 6B. This region spanned markers *Xgwm816* and *Xwmc397* and showed LOD values >3 in two experiments with both populations, but only the QTL in FTd was significant in individual experiments and in the multienvironment analysis. In addition, a QTL on chromosome 6A appeared in two experiments of population DTd. Finally, a QTL on 7B was significant in individual experiments of population FTd and HTd but not in the multienvironment analysis. This QTL showed peaks in both populations at *Xs24m12_f6h5* close to SSR marker *Xgwm400* and overlapped with a QTL for flowering time.

#### QTL analysis for developmental and morphological traits

Quantitative trait loci and estimates of QTL effects of plant height, spike compactness, length of awns and flowering date are shown in Table 5. Linkage groups and position of QTL are depicted in Electronic Supplementary Fig. S2.

**Table 5:** Summary of QTL for developmental and morphological traits identified by simple interval mapping

Trait	Population											
Chromosome	DTd				FTd				HTd			
	Flanking markers	Add^a^	PV %	LOD^b^	Flanking markers	Add^a^	PV %	LOD^b^	Flanking markers	Add^a^	PV %	LOD^b^
Plant height												
3A	–	–	–	–	*Xbarc67*–*Xgwm1121*	−6.6	14.5	8.1***	–	–	–	–
4B	*Rht*-*B1*–*Xs11m14_d1h1*	−17.3	68.5	58.7***	*Rht*-*B1*	−13.1	56.2	42.5***	*Rht*-*B1*	−18.4	67.8	59.0***
Spike compactness												
5A	*Xs20m15_d2*– *Xgwm179*	0.8	11.2	6.0***	*Xgwm179*–*Xgwm291*	0.7	10.7	5.7***	–	–	–	–
7A	–	–	–	–	*Xs11m24_f3*–*Xgwm681*	−0.6	8.9	4.7**	*Xs24m26_h6*–*Xgwm233*	−0.6	6.2	3.4**
Awn length												
3B	*Xs24m14_d9h6*–*Xs25m12_d9*	0.9	10.4	5.6*	–	–	–	–	–	–	–	–
4A	*Xgwm781*–*Xs24m12_d5*	1.5	29.7	17.9***	*Xgwm781*–*Xgwm610*	1.5	32.9	20.0***	*Xgwm781*–*Xs11m26_d9h4*	1.8	45.5	31.3***
7A	*Xs23m13_d13*–*Xgwm666a*	1.0	16.1	8.9***	*Xs11m24_f3*–*Xgwm681*	1.1	16.5	9.1***	*Xs24m26_h6*–*Xgwm233*	1.0	12.4	6.8***
Flowering date												
2B	–	–	–	–	*Xgwm429*–*Xgwm148*	−1.2	10.6	23***	–	–	–	–
4A	*Xs13m14_d10*–*Xs20m14_d2*	−0.6	6.2	13.1***	–	–	–	–	–	–	–	–
5A	*Xgwm179*–*Xs23m14_d16*	0.7	7.3	15.4***	–	–	–	–	–	–	–	–
7B	*Xs24m26_d16f4*	−1.0	10.8	23.2***	*Xs24m26_d16f4*–*Xgwm46*	−1.4	18.8	42.6***	*Xs23m12_h4*–*Xgwm46*	−1.1	13.1	29.1***

* α 0.1 < LOD; ** α 0.05 < LOD; *** α 0.01 < LOD

^a^Positive additive effects denotes that the *T.diccocum* allele reduces trait values relative to its respective *T. durum* allele

^b^Significance thresholds were estimated by permutation tests (number of iterations = 1,000) for α 0.01, α 0.05, α 0.1

Population DTd and HTd showed one, and population FTd two QTL associated with plant height. The *Rht*-*B1* QTL on 4B was significant in all populations. This QTL explained 56 % of PV in population FTd and 68 % in populations DTd and HTd. Lines homozygous for *Rht*-*B1b* allele were on average 25, 32, and 35 cm shorter compared to lines homozygous for the *Rht*-*B1a* wild-type allele in the respective populations FTd, DTd and HTd. Plant height in population FTd was influenced by a second QTL on 3A, which contributed 15 % to the PV and accounted for on average a 13 cm height difference between homozygous lines of contrasting allele status. The Td161 allele on 4B and 3A increased height.

Altogether four different genomic regions identified on chromosomes 2B, 4A, 5A, and on 7B affected the date of flowering. The strongest effect was from the QTL on 7B, which accounted for 11, 19, and 13 % of PV for DTd, FTd, and HTd, respectively. This QTL coincided with a minor QTL for FHB severity. The Td161 allele retarded flowering except at QTL on 5A.

Spike compactness was influenced by two QTL, assigned to chromosome 5A and 7A. Both QTL were identified in two populations, with the *T. durum* allele associated with compactness on 5A and laxness on 7A.

Three QTL on chromosomes 3B, 4A and 7A were associated with awn length. QTL on 4A and 7A were significant in all individual populations. The strongest effect was from the QTL on 4A which explained 30, 33, and 45 % PV in populations DTd, FTd and HTd, and a QTL on 7A contributed 16, 16, and 12 % to PV, respectively. At all these QTL the Td161 allele conferred reduced awn length.

## Discussion

By analyzing three back-cross populations between the FHB-resistant *T. dicoccum* donor line and three adapted *T. durum* varieties we combined QTL detection with QTL validation. The populations showed large genetic variation for FHB severity in the inoculated trials. The populations also segregated for plant morphological and developmental traits, such as plant height, awn length, spike morphology and flowering date.

Despite individual spray inoculation of each line followed by uniform mist irrigation, we found a negative correlation between FHB severity and plant height, as well as FHB severity and flowering date and a positive correlation between FHB severity and temperature at inoculation date. These results agree with previous reports. Particularly plant height has repeatedly been found associated with FHB severity measured in spray-inoculated experiments (e.g., Buerstmayr et al. 2000; Draeger et al. 2007; Holzapfel et al. 2008; Srinivasachary et al. 2009; Steiner et al. 2004). Variation in plant height in our populations was large. In view of differences in plant height between the tallest and shortest lines of up to 80 cm it is likely that heads of taller plants dried off faster, and were therefore under lower infection pressure than short plants even in the presence of mist irrigation intended to standardize humidity. Thus, at least part of the negative correlation between height and FHB severity could be due to plant height per se.

### QTL for Fusarium head blight resistance

Five QTL were found associated with FHB resistance, with *T. dicoccum* contributing the resistance-improving allele at four of these. FHB resistance in our populations is obviously under polygenic and complex genetic control. Three of four *T. dicoccum*-derived resistance QTL appeared in at least two populations, with only the minor QTL at chromosome 6A unique to one population. Interestingly, all detected QTL, except that on chromosome 6A, mapped to genomic regions previously associated with FHB resistance in hexaploid wheat.

3B: the *T. durum* cultivar Floradur contributed a resistance conferring QTL allele on chromosome 3BS close to *Xbarc133*. This QTL maps exactly to the position of the well-documented *Fhb1* (syn. *Qfhs.ndsu*-*3BS*) QTL from the cultivar Sumai-3 and other Asian resistance sources (Anderson et al. 2001; Liu et al. 2006, 2008; Waldron et al. 1999). To date, *Fhb1* has been found in more than 20 QTL mapping studies, all based on hexaploid Chinese resistance sources (Buerstmayr et al. 2009); this is the first report, where a resistance QTL at *Fhb1* was found in tetraploid wheat. Comparison of the allele size of SSR markers of the *Fhb1*-carrying hexaploid wheat cultivar Sumai-3 to alleles of the tetraploid wheat parents used in the present study with markers in close proximity to *Fhb1* suggests that the *Fhb1* allele of Sumai-3 is not identical to the allele in Floradur. Interestingly, although the SSR marker haplotype around *Fhb1* of Floradur was identical to that of Helidur, the 3B QTL was not detected in the Helidur population.

4B: by far the largest contribution to FHB severity was due to the QTL on 4B, which coincided with the *Rht*-*B1* locus. The FHB QTL at *Rht*-*B1* appeared in all populations, but its effect on FHB severity varied between populations. Since all populations were tested in the same environments, these differences can be attributed to their different genetic backgrounds or to sampling effects due to the relatively small population sizes. Notably, in *F. graminearum*-inoculated experiments with markedly lower average infection levels than in *F. culmorum* experiments the 4B QTL was always significant. In contrast, in the *F. culmorum*-inoculated experiments with high average FHB severity this QTL was significant only in population HTd. As resistance to FHB is non-species specific (Van Eeuwijk et al. 1995), this result suggests that above a certain infection pressure, the disease-reducing effect of increased height diminishes depending on the genetic background. In several independent studies in hexaploid wheat, the semi-dwarfing allele *Rht*-*D1b* was strongly associated with increased FHB severity (Draeger et al. 2007; Hilton et al. 1999; Holzapfel et al. 2008; Srinivasachary et al. 2008; Voss et al. 2008), but the association of the homeologous gene *Rht*-*B1* with FHB is less clear. *Rht*-*B1* and *Rht*-*D1* are orthologs of the *Arabidopsis Gibberellin*-*insensitive (GAI)* gene (Peng et al. 1999). Both genes exert, besides a strong effect on plant height, pleiotropic effects on various agronomic and quality traits (Elias and Manthey 2005). Miedaner and Voss (2008) compared Mercia-derived NILs (near-isogenic lines) carrying different *Rht* alleles. They found an increased FHB rating in the presence of the *Rht*-*B1b* allele, but the difference to *Rht*-*B1a* wild type was not significant. In a Soissons × Orvantis doubled-haploid population segregating for *Rht*-*D1* and *Rht*-*B1* only the *Rht*-*D1* locus was associated with FHB resistance, whereas in a study with Mercia and Maris Huntsman-derived NILs, both *Rht*-*B1b* and *Rht*-*D1b* decreased type 1 resistance while *Rht*-*B1b* increased type 2 resistance (Srinivasachary et al. 2008). Yan et al. (2011) reported increased FHB severity on short near-isogenic lines carrying different *Rht* alleles. Interestingly, the negative effect of most semi-dwarf alleles, including *Rht*-*B1b*, on type 1 resistance largely disappeared when the short isolines were physically elevated so that their spikes were positioned at the same height as those of their respective tall counterparts. This result indicated that the effect of stem-shortening alleles on increasing FHB susceptibility is due mainly to plant height per se. In view of this and the other prior findings, we speculate that the FHB-resistance-improving effect of the 4B QTL associated with the tall allele *Rht*-*B1a* in our study is due partly to plant height per se. This speculation warrants further investigation.

6B: the position of the 6B FHB QTL shared by the DTd and FTd populations matches that of the well-documented *Fhb2* QTL on 6BS. Resistance sources of *Fhb2* in hexaploid wheat are Sumai-3 and related lines (Buerstmayr et al. 2009; Cuthbert et al. 2006; Häberle et al. 2009; Löffler et al. 2009; Shen et al. 2003). Besides in hexaploid wheat, a QTL corresponding to *Fhb2* was reported in tetraploid wheat as well. Somers et al. (2006) mapped an FHB resistance QTL on 6BS in a doubled-haploid population of *T. durum* variety Strongfield × *T. carthlicum* variety Blackbird, which was clearly coincident with *Fhb2*. Improved resistance was contributed either by *T. carthlicum* in the Strongfield × Blackbird population, or as in the present study by *T. dicoccum*. The SSR allele survey at *Fhb2* revealed different haplotypes for the hexaploid resistance source Sumai-3 and the tetraploid resistant line *T. dicoccum.* The coincidence of QTL on chromosome 3B with *Fhb1* and on 6B with *Fhb2* suggests that genetic variation of FHB resistance at the *Fhb1* and *Fhb2* loci is not restricted to hexaploid wheat, but that resistance-improving alleles at these loci exist in some tetraploid wheat accessions as well.

7B: only a small impact on FHB resistance was attributed to chromosome 7BS, though it was significant in three experiments. This QTL overlapped with a QTL for flowering date. Several QTL mapping projects with hexaploid wheat populations identified association between FHB resistance factors and markers on chromosome 7BS. Schmolke et al. (2005) found in the Dream/Lynx population an overlap of FHB and heading date on this region of 7BS. Furthermore, minor QTL associated with FHB for this region were found in two independent studies with bread wheat (Jiang et al. 2007; Klahr et al. 2007), but no coinciding QTL for heading date were reported.

6A: an FHB resistance QTL on chromosome 6A near *Xgwm356* is reported here for the first time. This QTL was, however, detected in only one population (DTd) and accordingly is less attractive for resistance breeding.

Although the broad-sense heritability coefficients for FHB severity measured by AUDPC in the investigated populations were high, the percentage of phenotypic variance explained by QTL was only moderate to low and varied widely between the populations. Although the failure to find effective resistance in extensive screens of *T. durum* accessions for FHB reaction (Elias et al. 2005) suggested a general lack of resistance genes in the durum wheat gene pool, the resistance-improving allele on 3B was derived from the *T. durum* parent. Likewise Somers et al. (2006), Gladysz et al. (2007) and Ghavami et al. (2011) found QTL at which FHB resistance was contributed by the *T. durum* parent. This means that durum wheat does not necessarily lack resistance alleles. These findings, together with the observation that resistance QTL introgressed from hexaploid wheat into durum wheat improved resistance in only a few cases (own unpublished data), support the hypothesis that either most durum wheats possess suppressors that silence or reduce the effect of resistance-improving QTL (Stack et al. 2002, Garvin et al. 2009) or the D genome contributes resistance-inducing genes that are absent in durum wheat (Fakhfakh et al. 2011).

### QTL for morphological and developmental traits and their association with FHB resistance

#### QTL for flowering date

Altogether four different QTL were associated with flowering date. A QTL on 7B with strong effects on flowering date was found in all three populations. This flowering-date QTL overlapped with a QTL for FHB resistance, with later flowering associated with reduced FHB severity. Flood and Halloran (1983) reported the presence of an *Eps* (earliness per se) gene on chromosome 7B, Kuchel et al. (2006) mapped a photoperiod QTL, Lin et al. (2008) reported a major early flowering QTL, and Sourdille et al. (2000) detected two minor QTL with overlapping confidence intervals in this region related to earliness per se or photoperiod response. Several studies have found positive associations between early flowering and FHB severity (e.g., Buerstmayr et al. 2011; Gervais et al. 2003; Holzapfel et al. 2008; Paillard et al. 2004; Schmolke et al. 2005; Steiner et al. 2004). No systematic association between flowering date and FHB severity was found in a multi-environment evaluation of 56 lines derived from several European winter wheat mapping populations tested in five different countries over 2 years. Presumably, environment-specific factors, most likely the weather conditions around flowering and inoculation time, caused either positive, negative or no correlations (Buerstmayr et al. 2008). This is in agreement with our result, where there was a negative correlation between flowering time and FHB severity for means over all experiments, but this dependence was not consistent across all individual experiments. Out of four QTL for flowering date, only the QTL on 7B coincided with FHB severity. As mentioned above FHB resistance QTL on 7BS were found in different mapping projects. Thus, it appears likely, that the 7BS FHB resistance QTL effect does not rely on a pleiotropic effect of flowering date only.

#### QTL for plant height

Our results confirmed the large effect of the *Rht*-*B1b* allele on plant height, which segregated in all three populations. Only population FTd segregated for an additional plant height QTL on chromosome 3A. While *Rht*-*B1* was also associated with FHB severity, as discussed above, the 3A plant height QTL was not. Our finding is thus in agreement with the literature. Numerous studies observed a co-location of plant height QTL with FHB resistance QTL (Buerstmayr et al. 2011; Draeger et al. 2007; Gervais et al. 2003; Häberle et al. 2009; Paillard et al. 2004; Schmolke et al. 2005; Voss et al. 2008), as discussed above, but not all plant height QTL influenced FHB severity.

#### QTL for spike compactness

Two minor QTL associated with spike compactness were found. Although this trait was moderately correlated with FHB severity, none of the detected compactness QTL overlapped with QTL for FHB resistance.

#### QTL for awn length

*Triticum dicoccum*-161 had shorter awns than the *T. durum* parents. Awn length segregated in the populations and three QTL controlling this trait were found, with QTL on 4A and 7A being detected across all populations, but none of these was associated with FHB resistance QTL. The 4A QTL may correspond to the *Hd* (hooded) gene on 4A (Sears 1954; Rao 1981; Sourdille et al. 2002), while the awn length QTL at 7A is described here for the first time.

## Summary and conclusions

*Triticum dicoccum* line 161 has been confirmed as highly FHB resistant. Though the *T. dicoccum* × *T. durum*-derived mapping populations segregated for FHB resistance, only a few QTL were discovered, all of relatively small effect, mapping to chromosomes 3B, 4B, 6A, 6B and 7B. All but the 6A QTL mapped to genomic regions where FHB resistance QTL were previously found in hexaploid wheat, indicating that some FHB-resistance genes are common to tetraploid and hexaploid wheat. The resistance QTL of the largest effect mapped to chromosome 4B at the position of the *Rht*-*B1* plant height gene where the *T. dicoccum* allele enhanced FHB resistance and plant height. Selected moderately FHB-resistant experimental lines from this project are being used for further crossing and pyramiding FHB resistance into adapted durum wheat germplasm.

## Electronic supplementary material

Below is the link to the electronic supplementary material.

Supplementary material 1 (PDF 467 kb)

Supplementary material 2 (PPTX 197 kb)
