# Supplementary material for: Mapping of QTL for Fusarium head blight resistance and morphological and developmental traits in three backcross populations derived from Triticum dicoccum × Triticum durum
Source: Theor Appl Genet. 2012 Aug 25;125(8):1751–65. doi: 10.1007/s00122-012-1951-2 (PMC3493669; doi:10.1007/s00122-012-1951-2)
Supplement: Supplementary file 1 — Supplementary material 1 (PDF 467 kb) [file 122_2012_1951_MOESM1_ESM.pdf]

Electronic Supplementary Materials

Fig. S1 Typical heads of *T.dicoccum* line 161, *T. durum* cultivar Floradur and *T. durum* cultivar Helidur

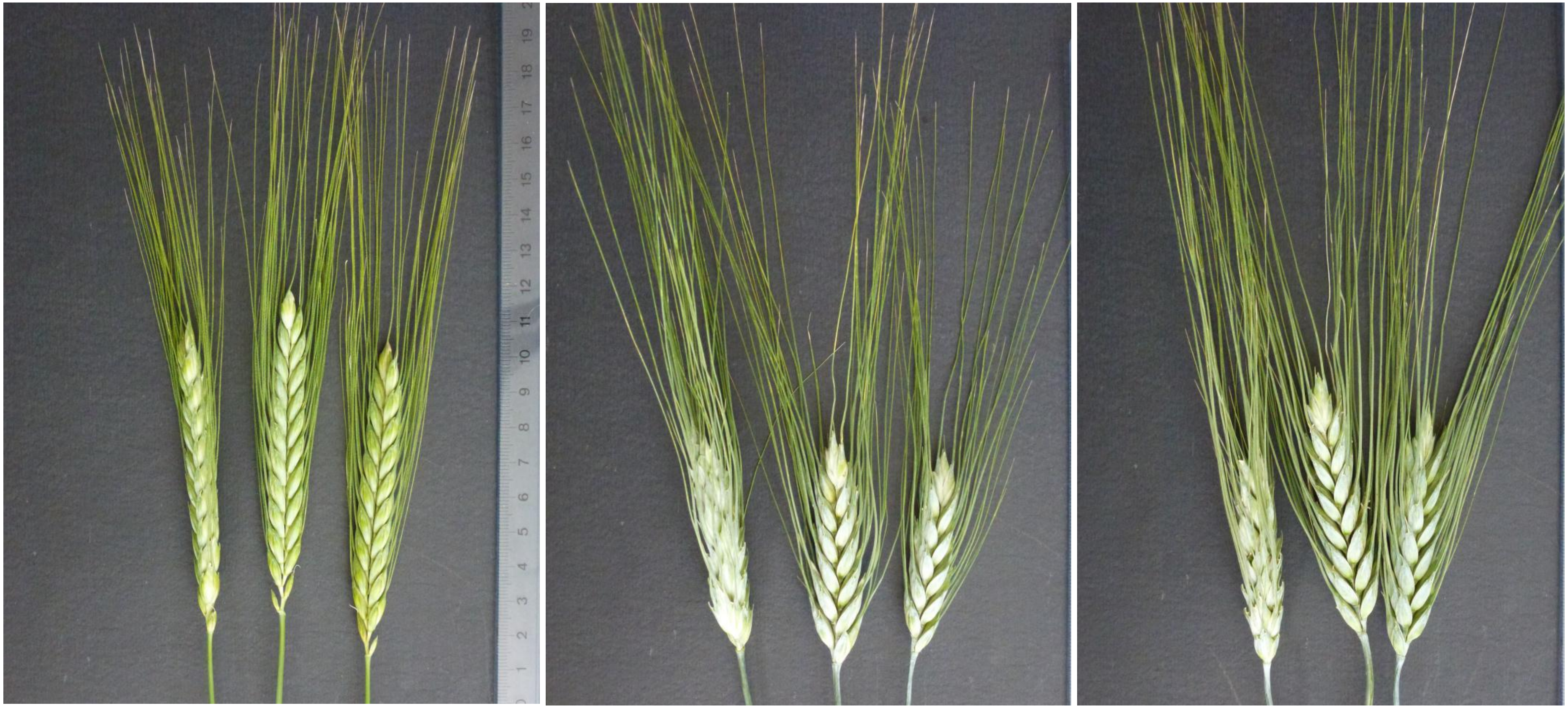

T. dicoccum - 161 *T. durum* Floradur *T. durum* Helidur
