# Supplementary material for: Mapping of QTL for Fusarium head blight resistance and morphological and developmental traits in three backcross populations derived from Triticum dicoccum × Triticum durum
Source: Theor Appl Genet. 2012 Aug 25;125(8):1751–65. doi: 10.1007/s00122-012-1951-2 (PMC3493669; doi:10.1007/s00122-012-1951-2)
Supplement: Supplementary file 2 — Supplementary material 2 (PPTX 197 kb) [file 122_2012_1951_MOESM2_ESM.pptx]

## Slide 1
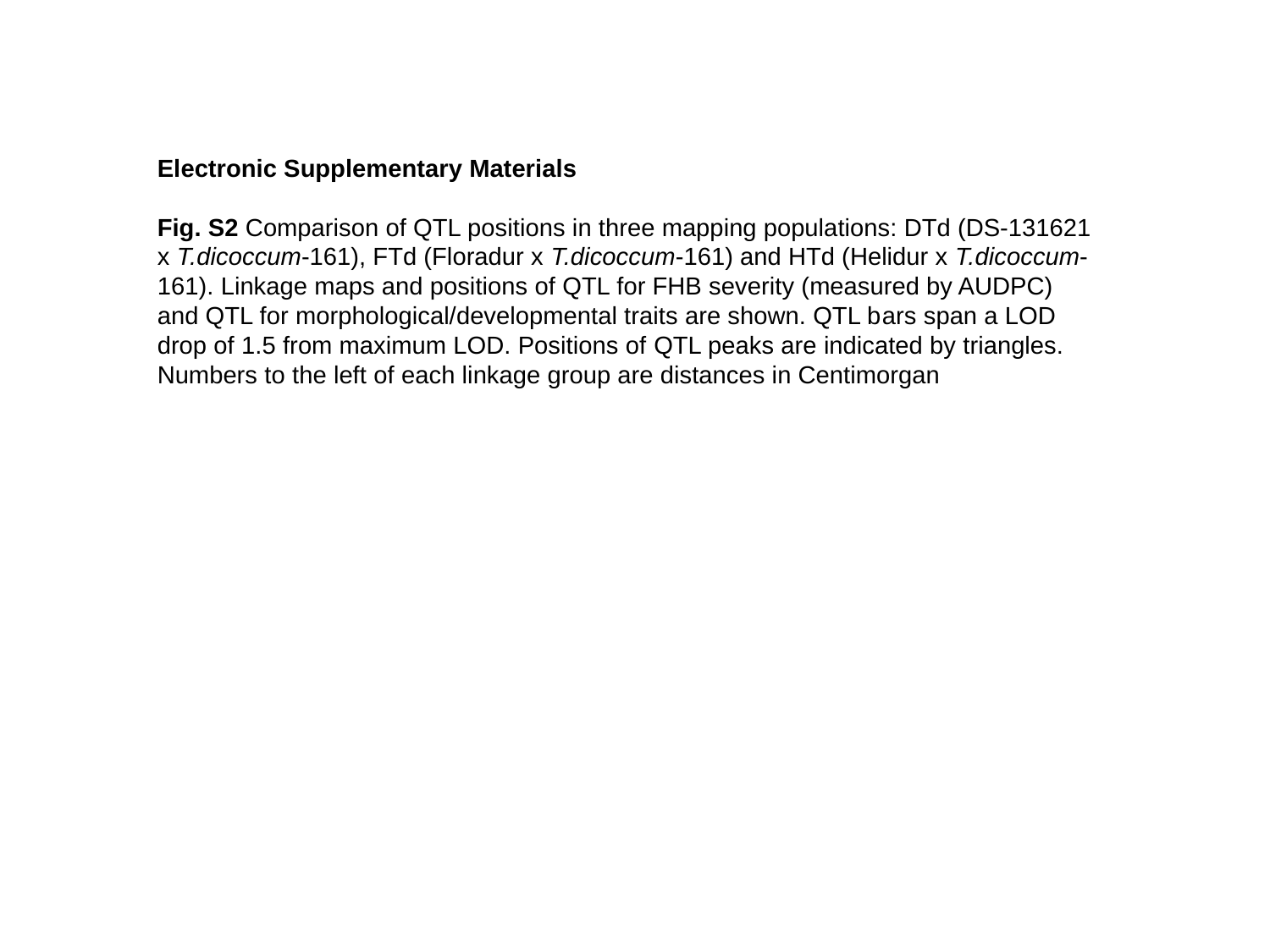

Electronic Supplementary Materials
Fig. S2 Comparison of QTL positions in three mapping populations: DTd (DS-131621 x T.dicoccum-161), FTd (Floradur x T.dicoccum-161) and HTd (Helidur x T.dicoccum-161). Linkage maps and positions of QTL for FHB severity (measured by AUDPC) and QTL for morphological/developmental traits are shown. QTL bars span a LOD drop of 1.5 from maximum LOD. Positions of QTL peaks are indicated by triangles. Numbers to the left of each linkage group are distances in Centimorgan

## Slide 2
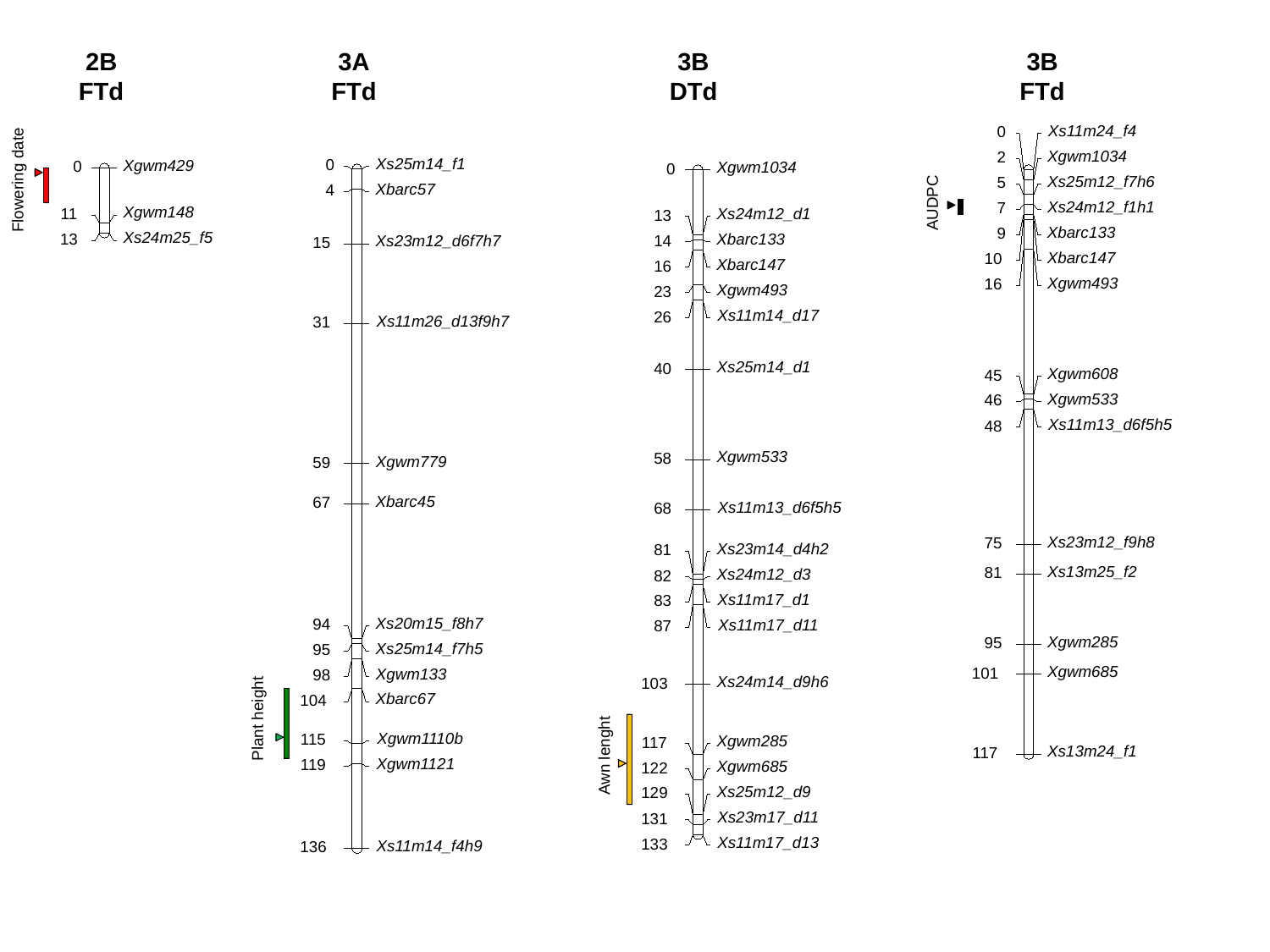

2B
FTd
Xgwm429
0
Flowering date
Xgwm148
11
Xs24m25_f5
13
3A
FTd
Xs25m14_f1
0
Xbarc57
4
Xs23m12_d6f7h7
15
Xs11m26_d13f9h7
31
Xgwm779
59
Xbarc45
67
Xs20m15_f8h7
94
Xs25m14_f7h5
95
Xgwm133
98
Xbarc67
104
Plant height
Xgwm1110b
115
Xgwm1121
119
Xs11m14_f4h9
136
3B
DTd
Xgwm1034
0
Xs24m12_d1
13
Xbarc133
14
Xbarc147
16
Xgwm493
23
Xs11m14_d17
26
Xs25m14_d1
40
Xgwm533
58
Xs11m13_d6f5h5
68
Xs23m14_d4h2
81
Xs24m12_d3
82
Xs11m17_d1
83
Xs11m17_d11
87
Xs24m14_d9h6
103
Xgwm285
117
Awn lenght
Xgwm685
122
Xs25m12_d9
129
Xs23m17_d11
131
Xs11m17_d13
133
3B
FTd
Xs11m24_f4
0
Xgwm1034
2
Xs25m12_f7h6
5
AUDPC
Xs24m12_f1h1
7
Xbarc133
9
Xbarc147
10
Xgwm493
16
Xgwm608
45
Xgwm533
46
Xs11m13_d6f5h5
48
Xs23m12_f9h8
75
Xs13m25_f2
81
Xgwm285
95
Xgwm685
101
Xs13m24_f1
117

## Slide 3
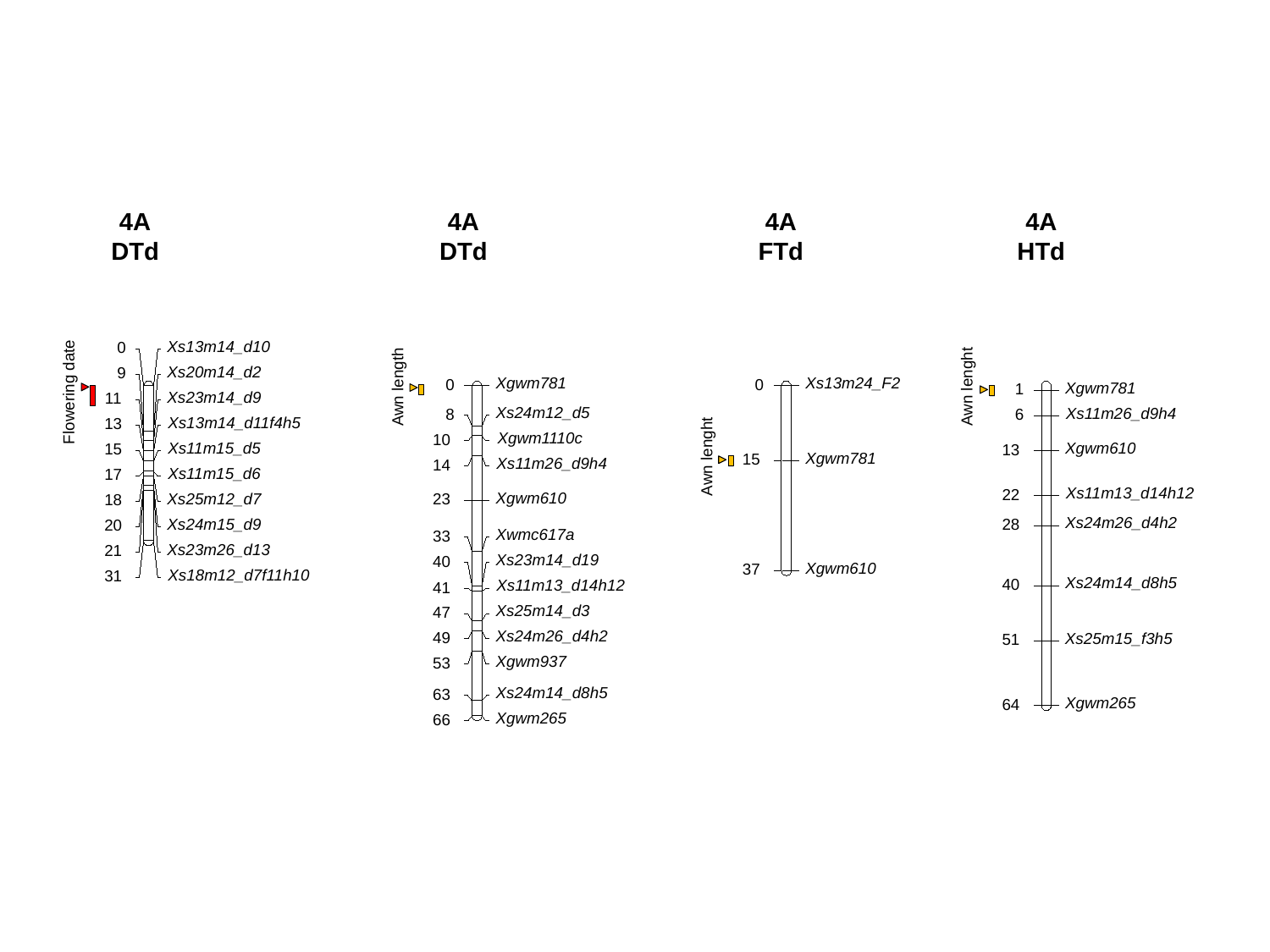

4A
DTd
Xs13m14_d10
0
Xs20m14_d2
9
Flowering date
Xs23m14_d9
11
Xs13m14_d11f4h5
13
Xs11m15_d5
15
Xs11m15_d6
17
Xs25m12_d7
18
Xs24m15_d9
20
Xs23m26_d13
21
Xs18m12_d7f11h10
31
4A
DTd
Xgwm781
0
Awn length
Xs24m12_d5
8
Xgwm1110c
10
Xs11m26_d9h4
14
Xgwm610
23
Xwmc617a
33
Xs23m14_d19
40
Xs11m13_d14h12
41
Xs25m14_d3
47
Xs24m26_d4h2
49
Xgwm937
53
Xs24m14_d8h5
63
Xgwm265
66
4A
FTd
Xs13m24_F2
0
Awn lenght
Xgwm781
15
Xgwm610
37
4A
HTd
Awn lenght
Xgwm781
1
Xs11m26_d9h4
6
Xgwm610
13
Xs11m13_d14h12
22
Xs24m26_d4h2
28
Xs24m14_d8h5
40
Xs25m15_f3h5
51
Xgwm265
64

## Slide 4
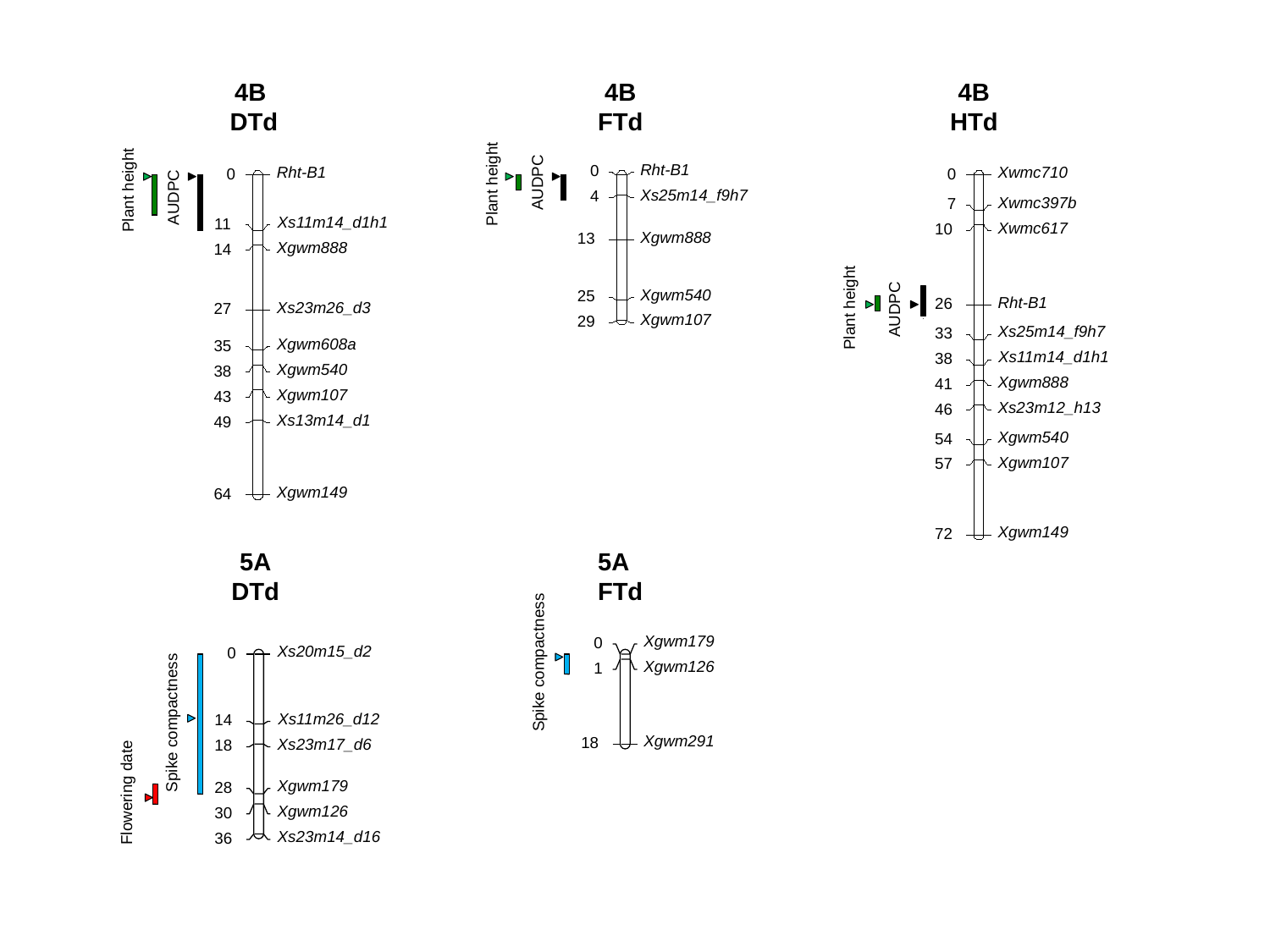

4B
DTd
Rht-B1
0
Plant height
AUDPC
Xs11m14_d1h1
11
Xgwm888
14
Xs23m26_d3
27
Xgwm608a
35
Xgwm540
38
Xgwm107
43
Xs13m14_d1
49
Xgwm149
64
4B
FTd
Rht-B1
0
AUDPC
Plant height
Xs25m14_f9h7
4
Xgwm888
13
Xgwm540
25
Xgwm107
29
4B
HTd
Xwmc710
0
Xwmc397b
7
Xwmc617
10
Rht-B1
26
Plant height
AUDPC
Xs25m14_f9h7
33
Xs11m14_d1h1
38
Xgwm888
41
Xs23m12_h13
46
Xgwm540
54
Xgwm107
57
Xgwm149
72
5A
DTd
Xs20m15_d2
0
Xs11m26_d12
14
Spike compactness
Xs23m17_d6
18
Xgwm179
28
Flowering date
Xgwm126
30
Xs23m14_d16
36
5A
FTd
Xgwm179
0
Spike compactness
Xgwm126
1
Xgwm291
18

## Slide 5
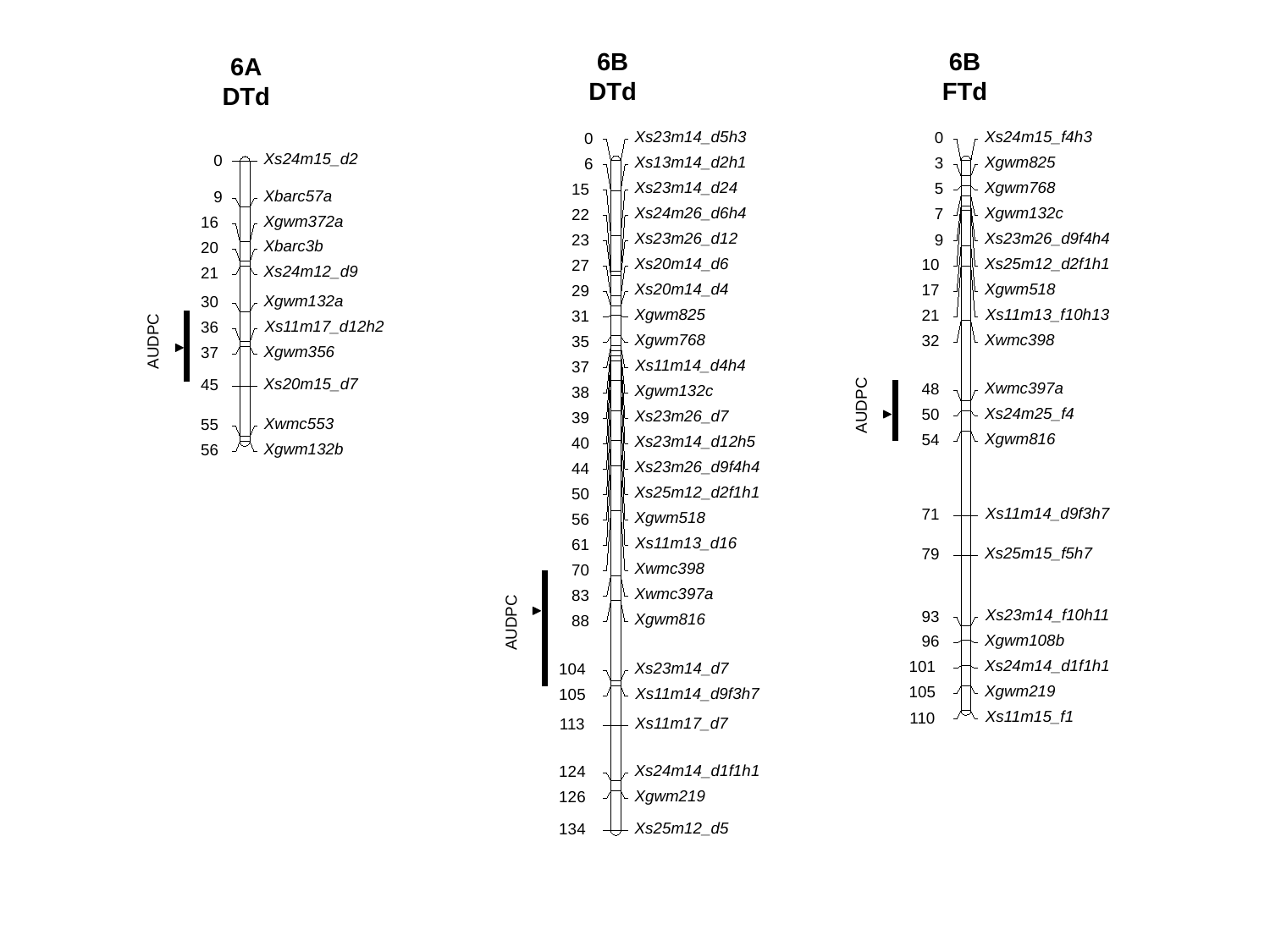

6B
DTd
Xs23m14_d5h3
0
Xs13m14_d2h1
6
Xs23m14_d24
15
Xs24m26_d6h4
22
Xs23m26_d12
23
Xs20m14_d6
27
Xs20m14_d4
29
Xgwm825
31
Xgwm768
35
Xs11m14_d4h4
37
Xgwm132c
38
Xs23m26_d7
39
Xs23m14_d12h5
40
Xs23m26_d9f4h4
44
Xs25m12_d2f1h1
50
Xgwm518
56
Xs11m13_d16
61
Xwmc398
70
Xwmc397a
83
Xgwm816
88
AUDPC
Xs23m14_d7
104
Xs11m14_d9f3h7
105
Xs11m17_d7
113
Xs24m14_d1f1h1
124
Xgwm219
126
Xs25m12_d5
134
6B
FTd
Xs24m15_f4h3
0
Xgwm825
3
Xgwm768
5
Xgwm132c
7
Xs23m26_d9f4h4
9
Xs25m12_d2f1h1
10
Xgwm518
17
Xs11m13_f10h13
21
Xwmc398
32
Xwmc397a
48
AUDPC
Xs24m25_f4
50
Xgwm816
54
Xs11m14_d9f3h7
71
Xs25m15_f5h7
79
Xs23m14_f10h11
93
Xgwm108b
96
Xs24m14_d1f1h1
101
Xgwm219
105
Xs11m15_f1
110
6A
DTd
Xs24m15_d2
0
Xbarc57a
9
Xgwm372a
16
Xbarc3b
20
Xs24m12_d9
21
Xgwm132a
30
Xs11m17_d12h2
36
AUDPC
Xgwm356
37
Xs20m15_d7
45
Xwmc553
55
Xgwm132b
56

## Slide 6
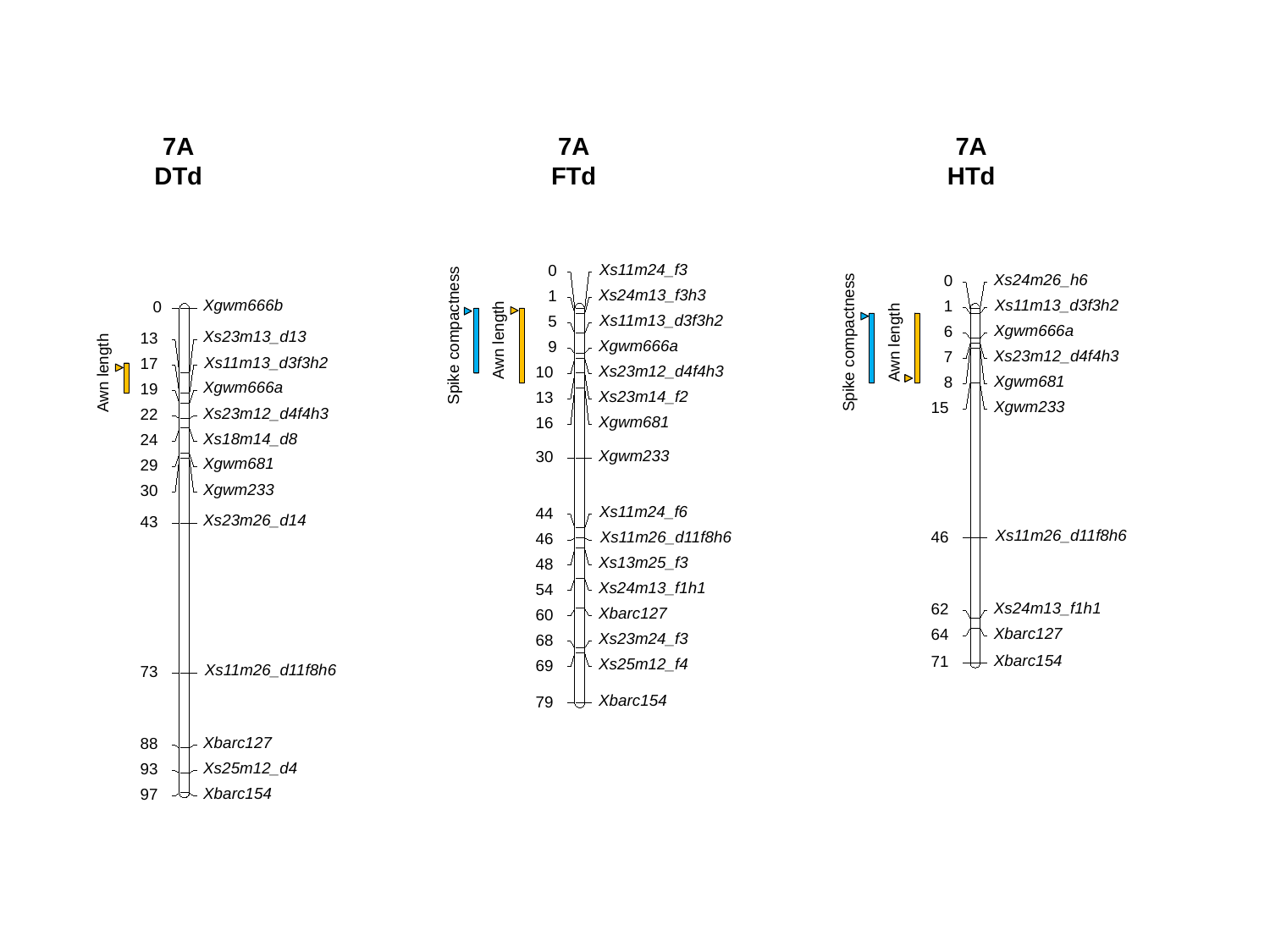

7A
DTd
Xgwm666b
0
Xs23m13_d13
13
Xs11m13_d3f3h2
17
Awn length
Xgwm666a
19
Xs23m12_d4f4h3
22
Xs18m14_d8
24
Xgwm681
29
Xgwm233
30
Xs23m26_d14
43
Xs11m26_d11f8h6
73
Xbarc127
88
Xs25m12_d4
93
Xbarc154
97
7A
FTd
Xs11m24_f3
0
Xs24m13_f3h3
1
Xs11m13_d3f3h2
5
Spike compactness
Awn length
Xgwm666a
9
Xs23m12_d4f4h3
10
Xs23m14_f2
13
Xgwm681
16
Xgwm233
30
Xs11m24_f6
44
Xs11m26_d11f8h6
46
Xs13m25_f3
48
Xs24m13_f1h1
54
Xbarc127
60
Xs23m24_f3
68
Xs25m12_f4
69
Xbarc154
79
7A
HTd
Xs24m26_h6
0
Xs11m13_d3f3h2
1
Xgwm666a
6
Spike compactness
Awn length
Xs23m12_d4f4h3
7
Xgwm681
8
Xgwm233
15
Xs11m26_d11f8h6
46
Xs24m13_f1h1
62
Xbarc127
64
Xbarc154
71

## Slide 7
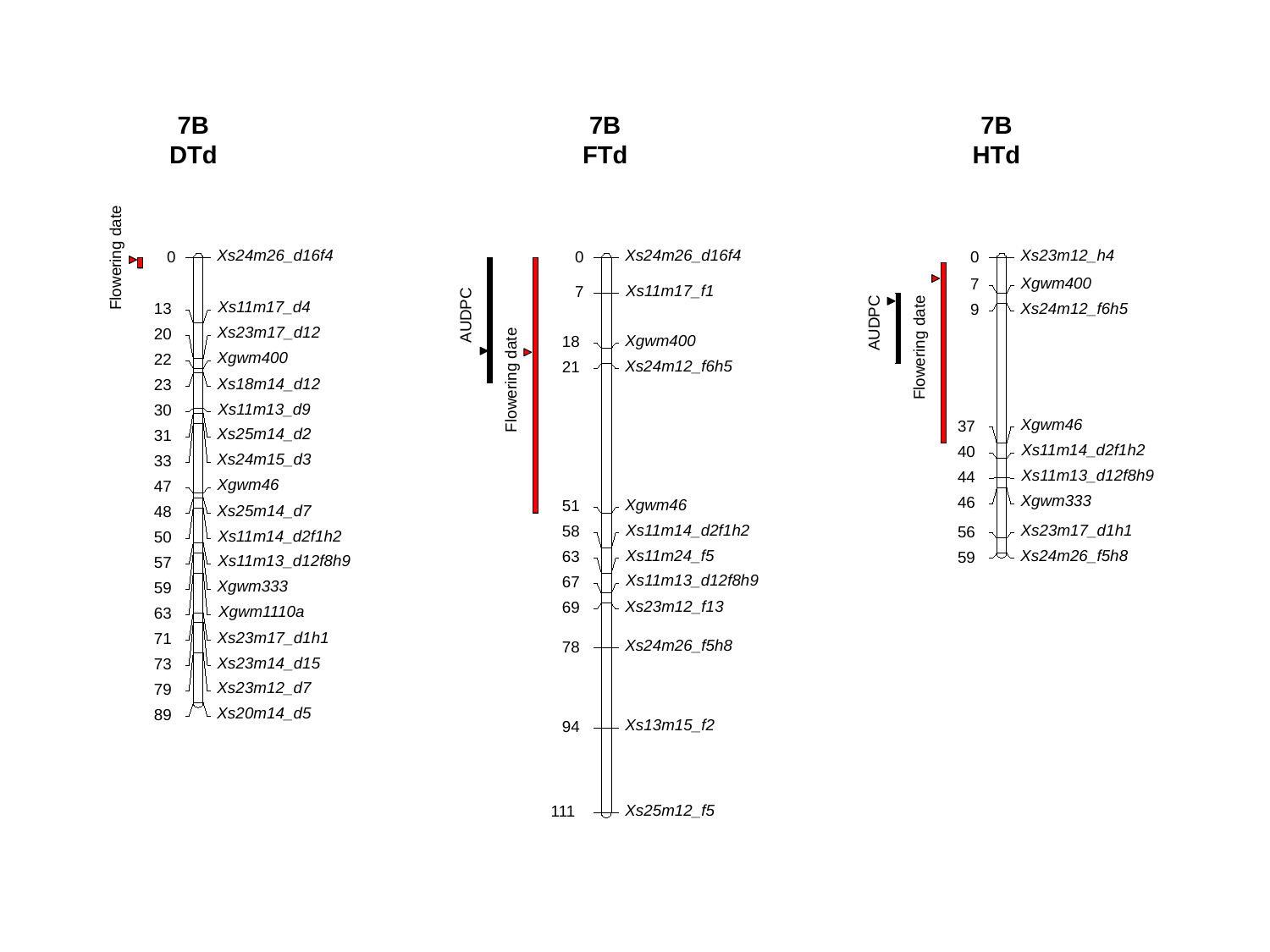

7B
DTd
Xs24m26_d16f4
0
Flowering date
Xs11m17_d4
13
Xs23m17_d12
20
Xgwm400
22
Xs18m14_d12
23
Xs11m13_d9
30
Xs25m14_d2
31
Xs24m15_d3
33
Xgwm46
47
Xs25m14_d7
48
Xs11m14_d2f1h2
50
Xs11m13_d12f8h9
57
Xgwm333
59
Xgwm1110a
63
Xs23m17_d1h1
71
Xs23m14_d15
73
Xs23m12_d7
79
Xs20m14_d5
89
7B
FTd
Xs24m26_d16f4
0
Xs11m17_f1
7
AUDPC
Xgwm400
18
Xs24m12_f6h5
21
Flowering date
Xgwm46
51
Xs11m14_d2f1h2
58
Xs11m24_f5
63
Xs11m13_d12f8h9
67
Xs23m12_f13
69
Xs24m26_f5h8
78
Xs13m15_f2
94
Xs25m12_f5
111
7B
HTd
Xs23m12_h4
0
Xgwm400
7
Xs24m12_f6h5
9
AUDPC
Flowering date
Xgwm46
37
Xs11m14_d2f1h2
40
Xs11m13_d12f8h9
44
Xgwm333
46
Xs23m17_d1h1
56
Xs24m26_f5h8
59
